# Effects of *Bacteroides*-Based Microecologics against Antibiotic-Associated Diarrhea in Mice

**DOI:** 10.3390/microorganisms9122492

**Published:** 2021-12-01

**Authors:** Hang Guo, Leilei Yu, Fengwei Tian, Jianxin Zhao, Hao Zhang, Wei Chen, Qixiao Zhai

**Affiliations:** 1 State Key Laboratory of Food Science and Technology, Jiangnan University, Wuxi 214122, China; guohang178@163.com (H.G.); edyulei@126.com (L.Y.); fwtian@jiangnan.edu.cn (F.T.); zhaojianxin@jiangnan.edu.cn (J.Z.); zhanghao61@jiangnan.edu.cn (H.Z.); chenwei66@jiangnan.edu.cn (W.C.); 2 School of Food Science and Technology, Jiangnan University, Wuxi 214122, China; 3 National Engineering Research Center for Functional Food, Jiangnan University, Wuxi 214122, China; 4 Wuxi Translational Medicine Research Center and Jiangsu Translational Medicine Research Institute Wuxi Branch, Wuxi 214122, China

**Keywords:** *Bacteroides*, *Bifidobacterium*, AAD, mice

## Abstract

Antibiotic-associated diarrhea (AAD) is a self-limiting disease mediated by antibiotic therapy. In clinical practice, several types of probiotics are used in treating AAD, but minimal research has been done on *Bacteroides*-based microecologics. Our aim was to evaluate the therapeutic effects of *Bacteroidetes uniformis* FGDLZ48B1, *B. intestinalis* FJSWX61K18, *Bifidobacterium adolescentis* FHNFQ48M5, and *B. bifidum* FGZ30MM3 and their mixture on AAD in mice. The lincomycin hydrochloride-induced AAD models were gavaged with a single strain or a probiotic mixture for a short period to assess the changes in colonic histopathology and cytokine concentrations, intestinal epithelial permeability and integrity, short-chain fatty acids (SCFAs), and the diversity of intestinal microbiota. Our data indicated that both the sole use of *Bacteroides* and the combination of *Bacteroides* and *Bifidobacterium* beneficially weakened systemic inflammation, increased the recovery rate of tissue structures, increased the concentrations of SCFAs, and restored the gut microbiota. Moreover, the probiotic mixture was more effective than the single strain. Specifically, *B. uniformis* FGDLZ48B1 combined with the *B. adolescentis* FHNFQ48M5 group was more effective in alleviating the pathological features of the colon, downregulating the concentrations of interleukin (IL)-6, and upregulating the expression of occludin. In summary, our research suggests that administration of a mixture of *B. uniformis* FGDLZ48B1 and *B. adolescentis* FHNFQ48M5 is an effective approach for treating AAD.

## 1. Introduction

Antibiotic-associated diarrhea (AAD) is a major clinical complication caused by side effects and the overuse of antibiotics [1,2]. AAD is estimated to typically occur in approximately 5–30% of patients during or at the end of antibiotic therapy [3], and often caused significant changes in gut microbiota [4]. Clinical symptoms in patients with AAD vary from mild diarrhea without complications to severe colitis, fulminant pseudomembranous colitis, or even death [5]. A possible mechanism of AAD is the antibiotic acting directly on the intestinal mucosa, thereby leading to the overgrowth of pathogenic bacteria such as *Clostridium difficile Staphylococcus*, *Candida*, *Enterobacteriaceae*, and *Klebsiella* [6]. Of these, *C. difficile* is the most common cause of AAD infections [7,8]. The use of antibiotics can cause dysregulation of the metabolic activity of colonic microbiota [9]. Microbial metabolites can modulate the metabolic integrity of epithelial cells and elicit immune reactions [10,11]. In addition, decreased resistance to pathogens caused by AAD is associated with alterations in the metabolism of carbohydrates, SCFAs, and bile acids [12].

Growing evidence indicates that AAD is caused by dysbiosis of the gut microbiota caused by antibiotic treatment [13,14]. Probiotics may maintain or recover intestinal microecology through nutritional competition, receptor competition, favoring the growth of nonpathogenic bacteria, inhibiting mucosal adhesion of pathogens, or simulating immunity during or after antibiotic treatment [15,16]. Current research is mainly focused on the genera *Lactobacillus*, *Bifidobacterium*, and *Saccharomyces*, which are commonly used to prevent or treat AAD [17]. *Bifidobacterium* preparations are increasingly being used in treating pediatric AAD. Pooled evidence from a systematic review, including five *Bifidobacterium* preparations from 30 trials, showed that *Bifidobacterium* preparations might be effective in preventing and treating pediatric AAD [18]. In addition, a study showed that long-term consumption of *Clostridium butyricum* in combination with a *B. infantis* mixture facilitated the recovery of gut microbiota and colonic tissue structure, which was a superior response to that observed in single strains in AAD treatments [19]. All of these studies have reinforced the potential role of probiotics in alleviating AAD.

*Bacteroides* are being actively researched as next-generation probiotics (NGPs) because of their potential benefits to human health. Some of these species can inhibit pathogenic bacteria colonization [20] and alleviate intestinal inflammation [21]. In a recent study, researchers determined that *B. fragilis* ZY -312 recovered epithelial cell organization and barrier functions through the ERK signaling pathway, thereby improving the abundance of specific commensal microbiota, and subsequently ameliorating diarrheal symptoms associated with AAD [22]. Moreover, the latest study correlated data from 117 individuals in four population-based cohorts and found that 21 bacteria species, including *Bacteroides intestinalis*, *B. uniformis*, *B. adolescentis*, and *B. bifidum*, showed a strong correlation with ecological recovery following antibiotic treatment [23]. In addition, a series of studies have demonstrated that *B. uniformis* can reduce the expression of acyl carrier proteins, thereby inhibiting increases in lipopolysaccharide (LPS)-induced proinflammatory cytokine levels, and beneficially reducing inflammation [24,25].

In addition, Kern Rei Chng et al. showed that combining *B. thetaiotaomicron* and *B. adolescentis* promoted a synergistic recovery of diversity and mucin enrichment in a mouse model associated with microbiome recovery following antibiotic treatment [23]. They explained that this synergistic effect was a result of the reconstruction of the food web in the intestinal microbial ecosystem, thereby forming a positive feedback pathway, with bacteria capable of colonization, for example, *B. thetaiotaomicron* provides an energy source for *B. adolescentis* that cannot colonize, which in turn produces more additional short-chain fatty acids to promote colonization. These findings provide new ideas for developing probiotics that help in the recovery of gut microbiota following antibiotic treatment. Therefore, the purpose of this study was to explore whether *B. intestinalis* FJSWX61K18, *B. uniformis* FGDLZ48B1, *B. adolescentis* FHNFQ48M5, and *B. bifidum* FGZ30MM3 help alleviate AAD and whether there is a synergistic feedback effect between these species. Specifically, this study assessed whether a combined treatment protects against lincomycin hydrochloride-induced intestinal injury, which is one of the causative mechanisms of AAD metabolic dysfunction.

## 2. Materials and Methods

### 2.1. Bacterial Strains and Culture

*B. intestinalis* FJSWX61K18, *B. uniformis* FGDLZ48B1, *B. adolescentis* FHNFQ48M5, and *B. bifidum* FGZ30MM3 (Table 1) were obtained from the culture collection of the Food Microbiology Department, Jiangnan University (Wuxi, China). *Bifidobacterium* spp. were cultured in a modified de Man, Rogosa, and Sharpe (mMRS) medium. *Bacteroides* spp. were cultured in a brain heart infusion (BHI) medium. All the strains were cultured in a Whitley DG250 anaerobic workstation.

### 2.2. Animal Experimental Design

Male SPF BALB/c adult mice (20–22 g) from a single breeding colony were provided by Zhejiang Vital River Laboratory Animal Technology Co. Ltd. (Beijing, China). They were placed in an air-conditioned room (21–25 °C) with a relative humidity of 40–60% and a 12 h light/dark cycle.

After seven days of adaptation, the mice were randomly assigned to nine groups of 10 mice each as follows (Table 2): a normal control group (Con), an antibiotic-associated diarrhea group (Mod), and treatment groups, including *B. intestinalis* FJSWX61K18 (Bi), *B. uniformis* FGDLZ48B1 (Bu), *B. adolescentis* FHNFQ48M5 (Ba), *B. bifidum* FGZ30MM3 (Bb), *B. intestinalis* FJSWX61K18 and *B. adolescentis* FHNFQ48M5 (Bi + Ba), *B. uniformis* FGDLZ48B1 and *B. adolescentis* FHNFQ48M5 (Bu + Ba), and *B. intestinalis* FJSWX61K18 and *B. bifidum* FGZ30MM3 (Bi + Bb). The mice except the Con group were gavaged with lincomycin hydrochloride (3 g/kg) twice daily at 8:00 a.m. and 20:00 p.m. for 3 days. All mice were assessed daily based on the scoring standard of diarrhea status from a previous study [26]. Feces were collected prior to execution, and fecal water content was measured using the freeze-drying method.

Finally, all the mice were anesthetized with isoflurane. The serum was separated from centrifuged blood samples (3000 rpm, 15 min) and frozen at −80 °C. Feces from the colon and cecum of each mouse were collected and frozen at −80 °C. A portion of each colon was fixed with paraformaldehyde (4%), and the remainder was stored at −80 °C.

### 2.3. Ethics Statement

All experiments involving animals were conducted in accordance with the ethical policies and procedures approved by the Committee of Ethics of Jiangnan University, China (Approval no. JN. No 20210615b1000718(185)). The use and care of laboratory animals were in accordance with the guidelines established by the European Community (Directive 2010/63/EU). We strove to maximize the health of the mice and reduce their suffering.

### 2.4. Histological Colon Observations

Colon tissue sectioning and staining methods were performed as described by Sun et al. [27]. The colon samples were fixed with 4% paraformaldehyde, then dehydrated with different levels of ethanol and embedded in paraffin. Next, the paraffin sections (5 μm) were deparaffinized and stained with hematoxylin and eosin. A pathological section scanner was used to capture images.

### 2.5. Biochemical Analyses of the Colon and Serum

The colon tissue was broken in cold saline and centrifuged (3000× *g*, 4 °C, 10 min). A BCA protein assay kit (Beyotime Biotechnology Inc., Shanghai, China) was used to determine the protein content of the supernatant. The levels of IL-1β, IL-6, IL-17, and TNF-α were measured using the corresponding ELISA kits (R&D Systems China Co., Ltd., Shanghai, China). Serum LPS levels were determined using ELISA assay kits (Shanghai Enzyme-linked Biotechnology Co., Ltd., Shanghai, China).

### 2.6. Analysis of SCFAs

All caecal contents of each mouse (20–50 mg) were collected and stored at −80 °C. The extraction and determination of butyric acid, acetic acid, propionic acid, and isobutyric acid were conducted using a previously described method [28]. Gas chromatography—mass spectrometry (Shimadzu Corporation, Japan) was used to analyze the concentrations of SCFAs.

### 2.7. Real-Time PCR Analysis

Total RNA from the colon tissue was extracted, and cDNA was synthesized using a RevertAid First Strand cDNA Synthesis Kit (Vazyme Biotech Co., Ltd.; Nanjing, China). Gene expressions of Mucin-2, occluding, and sodium-hydrogen exchange protein (NHE)3 were detected using real-time quantitative polymerase chain reaction [29]. mRNA expression was determined using a real-time quantitative PCR instrument (CFX Connect; Bio-Rad), as well as a universal iTaq SYBR green Supermix and associated primers (Table 3). The conditions were 40 cycles of 95 °C for the 30 s, 95 °C for 5 s, and 60 °C for 30 s. The β-actin gene was used as a reference gene for the calculation of quantitative expression of the target gene. The expression of genes was calculated using the 2^−ΔΔCt^ method with the control group as the base.

### 2.8. Preparation of Total DNAs and HIGH throughput Sequencing Analysis

Colon contents were used to extract total genomic DNA using the FastDNA^®^ Spin kit (MP Biomedicals Ltd., Santa Ana, CA, USA). Sequencing of the gut microbiota genomes and data processing were performed as described in recent studies [30,31].

### 2.9. Statistical Analysis

Data are expressed as the mean ± standard error of the mean (SEM). All results were analyzed using one-way analysis of variance (ANOVA), followed by Dunnett’s multiple comparisons test using GraphPad Prism software version v8.0.2(263).

## 3. Results

### 3.1. Effects of Different Treatments on Diarrhea Status Scores and Water Content in Feces

**Figure 1:**
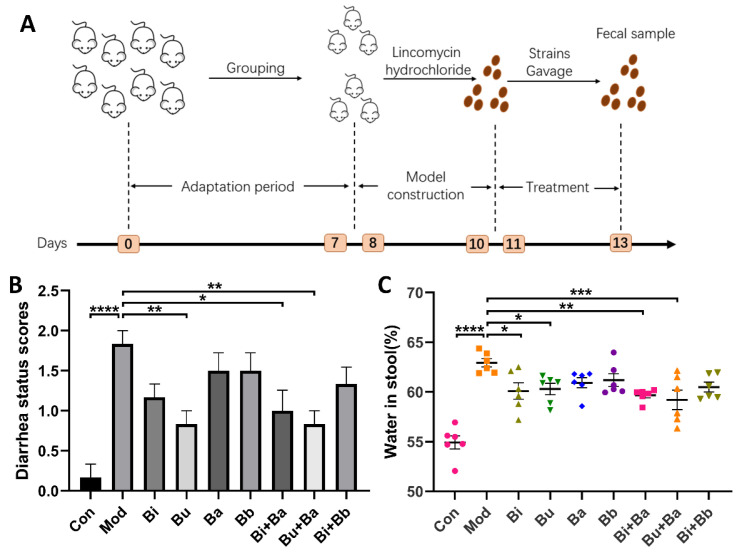
(**A**) Schematic diagram of the experimental design: (**B**) diarrhea status scores; and (**C**) water content in stool. *, **, ***, and **** indicate significant differences (*p* < 0.05, *p* < 0.01, *p* < 0.001, and *p* < 0.0001, respectively) between groups.

Initially, after the administration of lincomycin hydrochloride, all mice except the control group showed mental depression, a red anus, and diarrhea. Compared with the control group, the diarrhea status scores and stool water content of the model group was significant, which suggested that the AAD mouse model was successfully established. As shown in Figure 1B, a significant decrease in diarrhea status scores was observed in the Bu, Bi + Ba, and Bu + Ba groups. Among them, the Bu + Ba group showed a better result than the Bu group. Furthermore, the Bi + Ba and Bu + Ba groups exhibited significantly reduced stool water content. Although the Bi or Bu groups also showed a significant decrease, the effect was not as great as the mixed bacteria group (Figure 1C).

### 3.2. Effect of Different Treatments on Histopathological Structure of Colon Tissues

**Figure 2:**
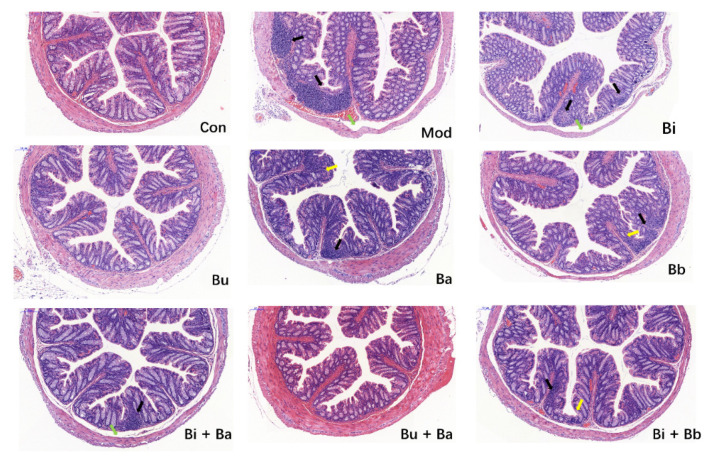
Histological examination of the representative image of H&E staining (scale bar = 100 µm). Yellow arrow—depletion of goblet cells; green arrow—mucosal edema; and black arrow—inflammatory cellular infiltration.

As shown in Figure 2, the colons of mice in the control group exhibited normal histological features. The mucosal epithelium remained intact, and the intestinal glands were abundant and closely arranged. Compared with the control group, the colonic mucosa of AAD mice showed a small inflammatory cell infiltration and mild edema in the submucosa. There was a slight reduction in the number of cupped glandular cells. These pathological characteristics were also observed in most treatment groups. However, no significant histopathological lesions were observed in mice treated with Bu or Bu + Ba, thereby indicating that it may reduce intestinal inflammation.

### 3.3. Effects of Different Treatments on Proinflammatory Cytokine Expression

**Figure 3:**
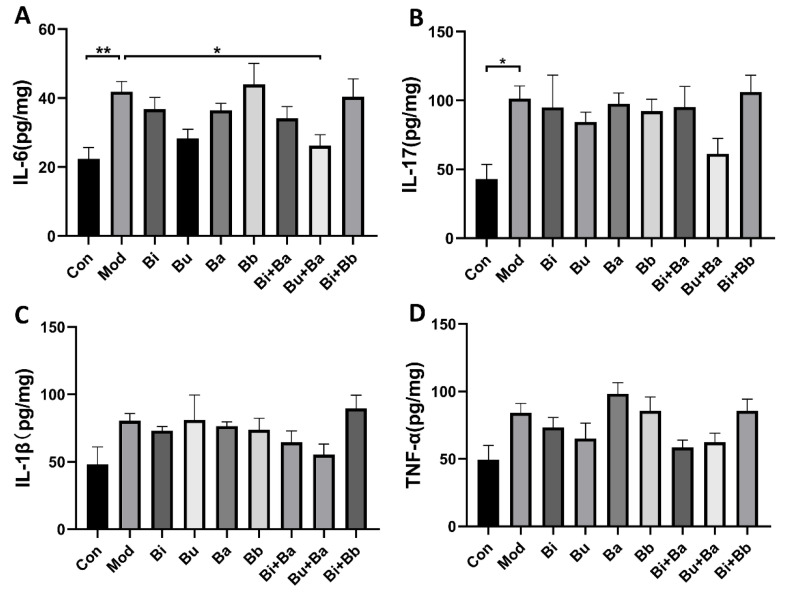
Analysis of cytokines in colon tissue by ELISA: (**A**) IL-6, (**B**) IL-17, (**C**) IL-1β, and (**D**) TNF-α. * and ** indicate significant differences (*p* < 0.05, *p* < 0.01 respectively) between groups.

To compare the immunomodulatory effects of different strains on AAD mice, cytokines were detected in colonic tissues by ELISA (Figure 3). The levels of IL-6 in the model group were significantly higher than those in the control group. Among the tested strains, the Bu + Ba group showed reduced levels of IL-6. Although the levels of IL-1β, IL-17, and TNF-α in the colonic tissues of mice were not significantly different from those in the control group, they showed a modest increase, whereas the Bu + Ba treatment decreased these proinflammatory inflammatory factors.

### 3.4. Effects of Different Treatments on Intestinal Barrier Integrity

**Figure 4:**
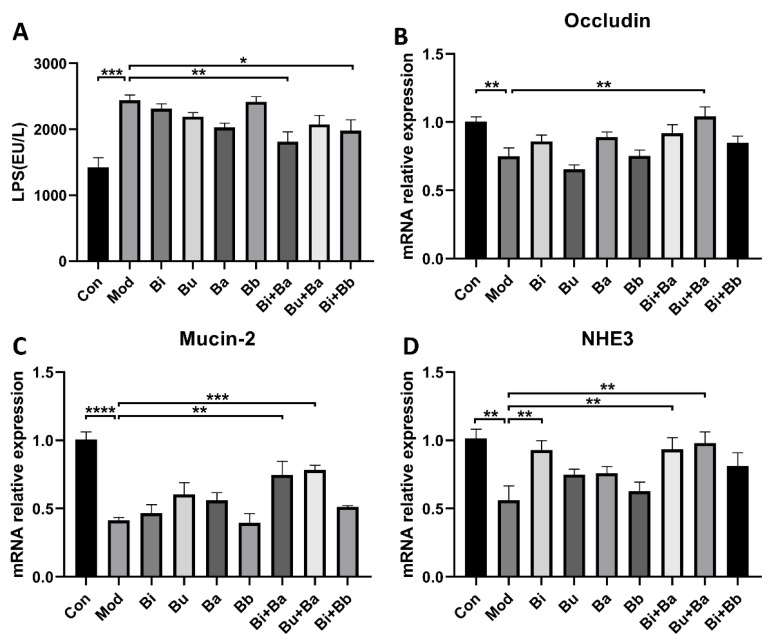
ELISA analyses of (**A**) lipopolysaccharide (LPS). Real-time PCR analysis of (**B**) occludin, (**C**) Mucin-2, and (**D**) NHE3, mRNA expression normalized to β-actin in the colons. *, **, ***, and **** indicate significant differences (*p* < 0.05, *p* < 0.01, *p* < 0.001, and *p* < 0.0001, respectively) between groups.

To analyze the regulation of different treatments on the intestinal barrier, serum LPS levels were determined. As shown in Figure 4, the level of LPS was significantly higher in the model group, whereas the Bi + Ba and Bi + Bb groups showed decreased LPS levels in the serum. Moreover, the Bi + Ba group showed a significant difference compared with the model group. Compared with the control group, the mRNA expression of occludin and Mucin-2 significantly decreased in the model group. However, the Bu + Ba treatment enhanced the expression of occludin in the colon to the level of the control group. In addition, Bi + Ba and Bu + Ba also increased the Mucin-2 expression. In the model group, diarrhea induced a significant decrease in the expression of NHE3 in the colonic tissues of the mice, whereas a supplementation with Bi, Bi + Ba, and Bu + Ba significantly increased this expression up to the level of the control group.

### 3.5. Effects of Different Treatments on SCFA Production

**Figure 5:**
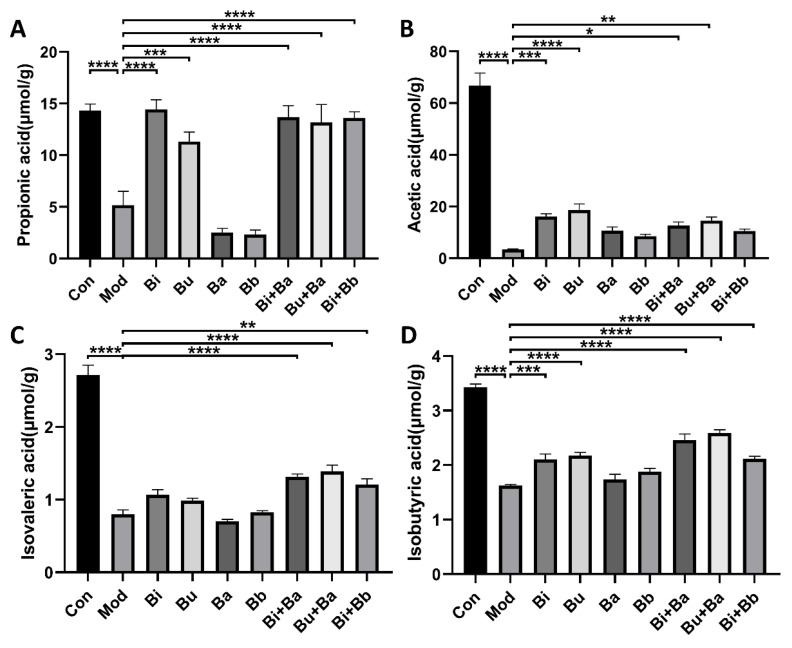
Effects of different treatments on the concentrations of (**A**) propionic acid, (**B**) acetic acid, (**C**) isovaleric acid, and (**D**) isobutyric acid. *, **, ***, and **** indicate significant differences (*p* < 0.05, *p* < 0.01, *p* < 0.001, and *p* < 0.0001, respectively) between groups.

After treatment with lincomycin hydrochloride, the concentrations of acetic acid, propionic acid, isobutyric acid, and isovaleric acid in the caecal specimens of the model group mice were significantly reduced (Figure 5). Compared with the model group, the Bi and Bu groups had significantly increased acetic acid, propionic acid, and isobutyric acid concentrations. The Bi + Ba treatment significantly increased propionic acid, isobutyric acid, and isovaleric acid, but did not significantly increase the level of acetic acid. Significant increases in acetic acid, propionic acid, isobutyric acid, and isovaleric acid concentrations were also observed in the Bu + Ba group. The Bi + Bb group showed significantly increased levels of propionic acid, isobutyric acid, and isovaleric acid in the caecal specimens. These results also indicated that the gavage of *Bacteroides* and the mixture of probiotics most strongly benefitted the recovery of SCFA concentrations in AAD mice.

### 3.6. Effect of Different Treatments on the Composition and Diversity of Gut Microbiota

**Figure 6:**
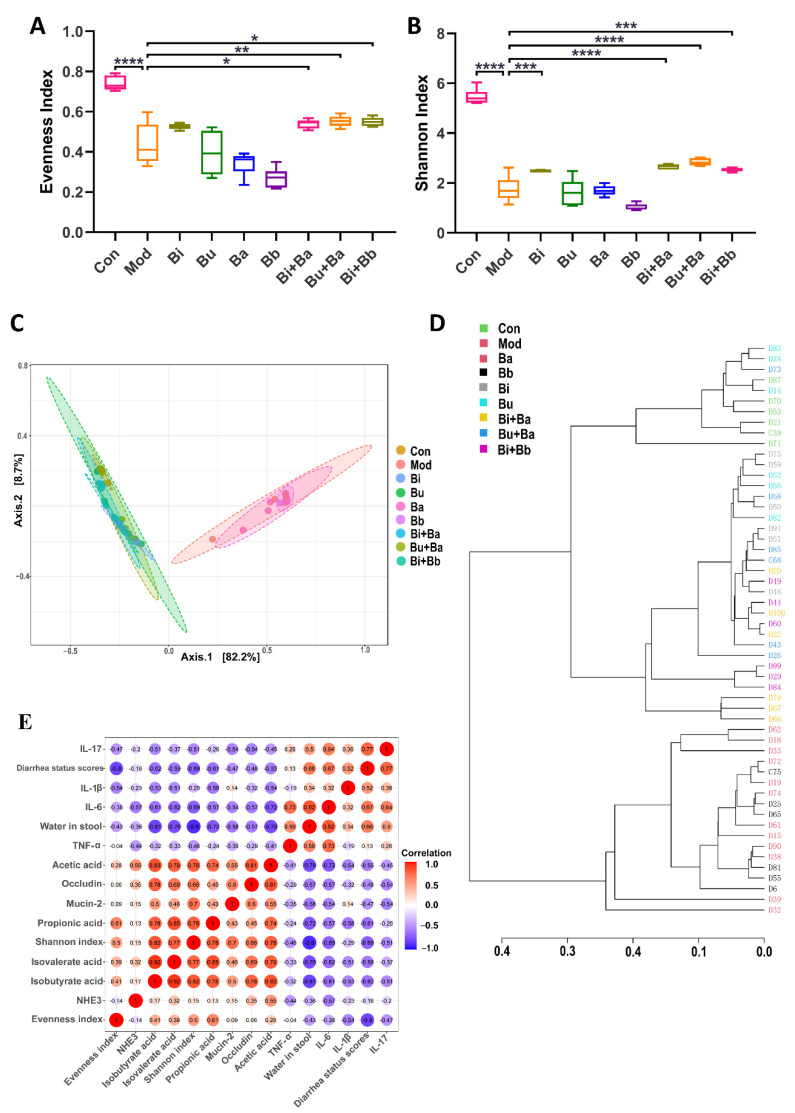
(**A**) Evenness index, (**B**) Shannon index, (**C**) principal coordinate analysis (PCoA), (**D**) clustering of distance based on the Jaccard index, and (**E**) correlation between the physiological and anti-inflammatory effects. *, **, ***, and **** indicate significant differences (*p* < 0.05, *p* < 0.01, *p* < 0.001, and *p* < 0.0001, respectively) between groups.

The evenness and Shannon indexes presented the evenness and diversity of the gut microbiota, respectively. The results showed that the evenness and diversity were significantly reduced following the lincomycin hydrochloride treatment (Figure 6A,B). Compared with the model group, the Bi + Ba, Bu + Ba, and Bi + Bb groups showed increased evenness index, and there was a significant difference in the increase in the Bu + Ba group. The highest Shannon index values were observed in the Bi + Ba and Bu + Ba groups. The Bi and Bi + Bb groups also exhibited a significant increase. The purpose of the β-diversity analysis was to determine the similarities among the groups, and the principal component analysis (PCoA) of community β-diversity and clustering of distance based on the Jaccard index analysis indicated that the clusters of the microbial compositions of the Bi, Bu, Bi + Ba, Bu + Ba, and Bi + Bb groups were closer to the control group than the model group; this indicated that all these treatment groups potently restored the intestinal microbiota disorder (Figure 6C,D). In conclusion, the mixture of *Bacteroides* and *Bifidobacterium* most positively impacted the recovery of flora diversity in AAD mice, with the Bu + Ba group facilitating the best recovery rate.

### 3.7. Spearman’s Correlation Analysis of Physiological and Anti-Inflammatory Effects

In this study, Spearman’s correlation coefficient was used to express the correlation between the two indicators. As shown in Figure 6E, the water content in the stool was negatively correlated with the Shannon index and the concentrations of isobutyric acid, and positively correlated with the levels of IL-6. In addition, the concentration of acetic acid was positively correlated with occludin. The evenness index was negatively correlated with the diarrhea status scores.

## 4. Discussion

This study aimed to determine whether *Bifidobacterium* strains, *Bacteroides* strains, and their mixture help alleviate AAD. We successfully established an AAD mouse model via gavage of lincomycin hydrochloride and explored the AAD-alleviating effects of these strains in animal models. The mice from the model group showed diarrhea-like symptoms and an increase in stool water content, as described in a previous study [26]. Notably, the establishment of this model resulted in mild signs of colonic inflammatory cell infiltration. These results suggested the induction of low-grade colonic inflammation in AAD mice. Our data demonstrated that *Bacteroides* and the combination of *Bacteroides* and *Bifidobacterium* alleviated the symptoms of AAD, and that the probiotic mixture groups facilitated the strongest recovery rate following AAD. The combination amplifies the benefits of probiotics to treat AAD, thereby enhancing our understanding of the potential mechanisms of bacterial interactions over the immune–metabolic axis.

In a previous study, the authors noted that the growth of some species depends on the presence of other species based on bacterial food webs [23]. We combined the selected strains based on the dependence patterns between microorganisms. During the recovery period following antibiotic treatment, some *Bacteroides* can more effectively colonize the epithelial mucosa owing to their mucin-degrading ability [32,33]. Because they also breakdown dietary carbohydrates of plant and animal origin, they act as keystone species to promote the growth of other species [34]. Other species, such as *Bifidobacterium*, can use the monosaccharides produced by degradation to grow and produce large amounts of SCFAs, which promote the growth of colon cells and thus increase mucin production [35]. From the perspective of diversity and biomass, this positive feedback loop may lead to a faster ecological recovery. In addition, the combined treatment may modify the metabolic pathways of microorganisms, thereby, altering the nutrients available in the intestinal lumen and improving their accessibility to the host. Our results confirmed that their combination reduced diarrhea status scores and water content in stools. This was accompanied by an improvement in energy metabolic pathways and the restoration of intestinal immune homeostasis [36]. Notably, *Bacteroides* play a greater role than *Bifidobacterium* in restoring metabolic alterations.

Changes in cytokines have also been associated with the use of antibiotics, such as tumor necrosis factor and interleukins, both of which act as communicators between immune cells and mirror the inflammatory profile of the host. As noted above, the colonic tissue showed mild edema and inflammatory infiltration in AAD mice. Therefore, we next assessed the levels of inflammatory cytokines in colonic tissue and found increases in the concentrations of inflammatory factors, including IL-6 and IL-17. Excessive amounts of proinflammatory cytokines can cause disturbances in the immune response, which in turn can lead to an inflammatory response [37]. The Bu + Ba group decreased the production of IL-6 to relieve intestinal inflammation. This result was consistent with a previous report [19], in which *C. butyricum* combined with the *B. infantis* probiotic mixture inhibited the AAD-induced inflammatory response by balancing the levels of proinflammatory cytokines and anti-inflammatory cytokines.

Compared with normal control animals, AAD mice exhibited defective gastrointestinal integrity and abnormal tight junction protein expression. These findings indicate that the defective intestinal barrier provides pathogens with access to the host, and this is likely the primary cause of diarrhea in AAD mice [22]. Occludin and Mucin-2 are two important intestinal barrier-related genes. Our results indicated that the mixture of probiotics restored their expression, and maintained the integrity and permeability of the intestinal barrier. Studies have shown that epithelial cells are surrounded by mucus and tight junctions of the gastrointestinal tract, known as the first barrier of the intestine [38]. Probiotics have beneficial effects in improving the barrier function of the intestinal mucosa. In addition, a previous study showed that a daily gavage of 10^9^ CFU of *B. fragilis* ZY-312 was associated with increases in mucin synthesis, ZO-1, and epithelial cell proliferation in the colon [22]. NHE3 is one of five plasma membrane Na+/H+ exchangers and plays an important role in fluid reabsorption and acid-base balance [39]. *B. subtilis* CU1 may increase the ability of the colon to absorb excessive water in the presence of diarrhea by acting on the expression of NHE3 [40]. In our study, treatment with *Bacteroides* stains increased the expression of NHE3 and thus promoted fluid absorption. Owing to the critical role of intraluminal solute concentration in the development of diarrhea, using probiotics to facilitate changes in intestinal electrolyte transporters may be an effective mechanism for treating AAD.

In this study, 16S rRNA sequencing was performed to determine the microbial composition of the mouse guts. Increases in the evenness index and Shannon index showed that *Bacteroides* and a mixture of probiotics enhanced the diversity of the microbial community. β-Diversity and distance clustering indicated that the lincomycin hydrochloride treatment significantly altered the overall structure of the microbial community. The mixture of probiotics and *Bacteroides* attenuated these changes, which resulted in a structure similar to that of the control group, as well as improved the intestinal lumen environment. The multiple effects of antibiotics on the gut microbiota include a reduction in the diversity and evenness of gastrointestinal microorganisms [41]. These drastic changes lead to the exhaustion of the normal intestinal microbiota residents and the chances for pathogens to colonize. Probiotics can inhibit intestinal pathogens by producing antimicrobial compounds, compete for rejection by consuming limited nutritional resources or adhering to epithelial cells, or stimulate intrinsic microbial activity [42]. Zhang et al. [22] observed overgrowths of *Klebsiella* and *Enterobacter* in an AAD rat model and demonstrated that an oral treatment with the *B. fragilis* strain ZY -312 alleviated antibiotic-associated syndromes by restoring intestinal microbiota diversity.

The majority of intestinal SCFAs originated from the fermentation and catabolism of indigestible carbohydrates by microorganisms in the colon via different pathways. SCFAs have reportedly been associated with the preservation of intestinal homeostasis. Acetic acid has been demonstrated to be critical in inhibiting intestinal pathogens [43], and the production of butyric acid may lead to an increase in mucin production and promote tight junction integrity [44]. The study also detailed a strong correlation between SCFA levels in feces and the abundance of *Bacteroides* [45]. The genus *Bacteroides* exhibits a high degree of adaptability to the nutritional requirements of the intestinal environment, and can use dietary or host-derived glycans depending on nutritional utilization [46]. Thus, they facilitate the growth of other species that can produce SCFAs [47]. As shown in Figure 5, our results confirmed this view again, and showed that the mixture of *Bacteroides* and *Bifidobacterium* most strongly improved restoration. The inhibitory effect of SCFAs on the production of proinflammatory mediators by neutrophils downregulates the proinflammatory cytokines [48]. SCFAs have reportedly inhibited the production of TNF-α, IL-2, and IL-1β by modulating the NF-κB pathway, thus resisting host inflammation [49]. Therefore, the high concentrations of SCFAs in the Bu + Ba group could be a potential mechanism for protecting against intestinal inflammation in AAD mice. We further analyzed the correlation between the mitigating effects of SCFAs and AAD, and the results validated our conclusions.

Unlike *Lactobacillus* and *Bifidobacterium*, whose entire species are Generally Regarded as Safe (GRAS) in the USA or licensed for consumption by the European Food Safety Authority [50], the health-promoting properties of *Bacteroides* are strongly dependent on the strain. Although several studies have considered *Bacteroides* as potential candidates for next-generation probiotics, the roles of different *Bacteroides* species in human health and disease are controversial. Researchers have found that *B. fragilis* containing virulent fragilysin genes accelerates inflammation [51]. Additionally, *B. fragilis* YCH46 produces fibrinogen-degrading proteases that may disrupt defense systems and enhance infection through a bacterial invasion of the injured tissue [52]. To date, only *B. xylanisolvens* DSM 23964 has been authorized by the European Commission to supplement pasteurized milk products according to the Novel Food Regulation No. 258/97 [53]. Therefore, it is necessary to conduct a comprehensive safety evaluation of *Bacteroides* and justify its use under the precondition of avoiding its pathogenicity. However, we also enabled a rational manipulation of the abundance of *Bacteroides* via diet. Previous studies have indicated that alginate from *Laminaria japonica* increases the relative abundance of *Bacteroides* species. Among them, *B. finegoldii* responded more positively to the intervention [54]. Another study elucidated the basis for proliferating of *Bacteroides* in response to fructans, and revealed a fructose-binding hybrid two component signaling sensor that controlled the fructose utilization site in *B. thetaiotaomicron* [55]. In summary, we suggest that further attention should be paid to screening and safety assessments of *Bacteroides* and the interactions between *Bacteroides* and other intestinal microorganisms and dietary factors.

## 5. Conclusions

Collectively, *Bacteroides* and the mixture of *Bifidobacterium* and *Bacteroides* protected against antibiotic-associated diarrhea in our study. *B. uniformis* FGDLZ48B1 + *B. adolescentis* FHNFQ48M5 had the best protective effect against diarrhea in mice with AAD. Specifically, Bu + Ba decreased the levels of inflammatory cytokines and increased the production of SCFAs, and then restored the integrity of the intestinal barrier by promoting the expression of tight junction proteins. Furthermore, Bu + Ba also changed the composition and structure of the intestinal microbiota of mice, thereby promoting the specimens’ recovery from AAD. However, additional studies are needed to evaluate the safety of *B. uniformis* FGDLZ48B1 in the human environment.

## Figures and Tables

**Table 1:** Strains used in the work.

Strain	Origin	Region
FJSWX61K18	Human feces	Jiangsu Province, China
FGDLZ48B1	Human feces	Guangdong Province, China
FHNFQ48M5	Human feces	Henan Province, China
FGZ30MM3	Human feces	Guizhou Province, China

**Table 2:** Animal experimental design.

Groups	Modeling Period (3 Days)	Recovery Period (3 Days)
Con	PBS	PBS
Mod	lincomycin hydrochloride (3 g/kg) twice daily	PBS
Bi	lincomycin hydrochloride (3 g/kg) twice daily	5 × 10^8^ CFUs of *B. intestinalis* FJSWX61K18
Bu	lincomycin hydrochloride (3 g/kg) twice daily	5 × 10^8^ CFUs of *B. uniformis* FGDLZ48B1
Ba	lincomycin hydrochloride (3 g/kg) twice daily	5 × 10^8^ CFUs of *B. adolescentis* FHNFQ48M5
Bb	lincomycin hydrochloride (3 g/kg) twice daily	5 × 10^8^ CFUs of *B. bifidum* FGZ30MM3
Bi + Ba	lincomycin hydrochloride (3 g/kg) twice daily	5 × 10^8^ CFUs of *B. intestinalis* FJSWX61K18 + 5 × 10^8^ CFUs of *B. adolescentis* FHNFQ48M5
Bu + Ba	lincomycin hydrochloride (3 g/kg) twice daily	5 × 10^8^ CFUs of *B. uniformis* FGDLZ48B1 + 5 × 10^8^ CFUs of *B. adolescentis* FHNFQ48M5
Bi + Bb	lincomycin hydrochloride (3 g/kg) twice daily	5 × 10^8^ CFUs of *B. intestinalis* FJSWX61K18 + 5 × 10^8^ CFUs of *B. bifidum* FGZ30MM3

**Table 3:** Primer sequences used for qPCR analysis.

Gene	Forward (5′ to 3′)	Reverse (5′ to 3′)
β-actin	GGCTGTATTCCCCTCCATCG	CCAGTTGGTAACAATGCCATGT
Mucin-2	CAACAAGCTTCACCACAATCTC	CAGACCAAAAGCAGCAAGGTA
Occludin	CACACTTGCTTGGGACAGAG	TAGCCATAGCCTCCATAGCC
NHE3	TGGCCGGGCTTTCGACCACA	GGGACCCACGGCGCTCTCCCT

## Data Availability

All data presented in this study are available in the main body of the manuscript.
